# Inflammatory arthritis in HIV positive patients: A practical guide

**DOI:** 10.1186/s12879-016-1389-2

**Published:** 2016-03-01

**Authors:** T. Adizie, R. J. Moots, B. Hodkinson, N. French, A. O. Adebajo

**Affiliations:** Rheumatology Department, Heart of England NHS Trust, Birmingham, UK; Rheumatology Department, Aintree University Hospital, Liverpool, UK; Clinical Sciences Centre, Aintree University Hospital, Longmoor Lane, Liverpool, L9 7AL UK; Department of Medicine, Groote Schuur Hospital and University of Cape Town, Cape Town, South Africa; Department of Clinical Infection, Microbiology & Immunology, Institute of Infection & Global Health, The University of Liverpool, Liverpool, UK; Academic Rheumatology Group, Faculty of Medicine, University of Sheffield, Sheffield, UK

**Keywords:** HIV, Acquired immune-deficiency syndrome, Rheumatic manifestations, Arthritis, Spondyloarthropathy, anti-TNF and Disease modifying antirheumatic drugs

## Abstract

**Background:**

Musculoskeletal manifestations of the human immunodeficiency virus (HIV) have been described since the outset of the global HIV epidemic. Articular syndromes that have been described in association with HIV include HIV-associated arthropathy, seronegative spondyloarthropathies (SPA) (reactive arthritis, psoriatic arthritis (PsA) and undifferentiated SPA), rheumatoid arthritis (RA) and painful articular syndrome.

**Methods:**

We carried out a computer-assisted search of PubMed for the medical literature from January 1981 to January 2015 using the keywords HIV, acquired immune-deficiency syndrome, rheumatic manifestations, arthritis, spondyloarthropathy, anti-TNF and disease modifying antirheumatic drugs. Only English language literature was included and only studies involving adult human subjects were assessed.

**Results:**

There are challenges in the management of inflammatory arthritis in patients who are HIV-positive, including difficulties in the assessment of disease activity and limited information on the safety of immunosuppressive drugs in these individuals.

**Conclusions:**

This review focuses on the clinical characteristics of the inflammatory articular syndromes that have been described in association with HIV infection and discusses the therapeutic options for these patients.

## Background

Musculoskeletal manifestations of the human immunodeficiency virus (HIV) have been described since the outset of the global HIV epidemic. The first reports of rheumatological symptoms of the infection occurred 3 years after its discovery, with Winchester et al. describing a case of reactive arthritis in a patient with advanced acquired immunodeficiency syndrome (AIDS) [1]. Substantial gains have been made at stemming the spread of HIV, with the rate of new cases each year steadily declining and the number of AIDS-related deaths also falling from 3.1 million in 2005 to 1.7 million in 2012 [2]. HIV positive patients are also living longer due to antiretroviral (ARV) therapy and as a result, chronic non-communicable diseases in long-term sufferers of HIV are now emerging as a significant cause of morbidity. Patients infected with HIV have been shown to have a higher risk of developing rheumatic diseases [3], and this can occur at any stage of the disease. Moreover, HIV positive patients having musculoskeletal involvement have reduced quality of life, when compared to those without rheumatic symptoms [4]. Articular syndromes that have been described in association with HIV include HIV-associated arthropathy, seronegative spondyloarthropathies (SPA) (reactive arthritis, psoriatic arthritis (PsA) and undifferentiated SPA), rheumatoid arthritis (RA) and painful articular syndrome. Other nonarticular rheumatological conditions including osteonecrosis, vasculitis and myositis are well described manifestations of HIV but are not within the scope of this article. This review focuses on the clinical characteristics of the inflammatory articular syndromes that have been described in association with HIV infection and discusses the therapeutic options for these patients.

## Methods

**Fig. 1:**
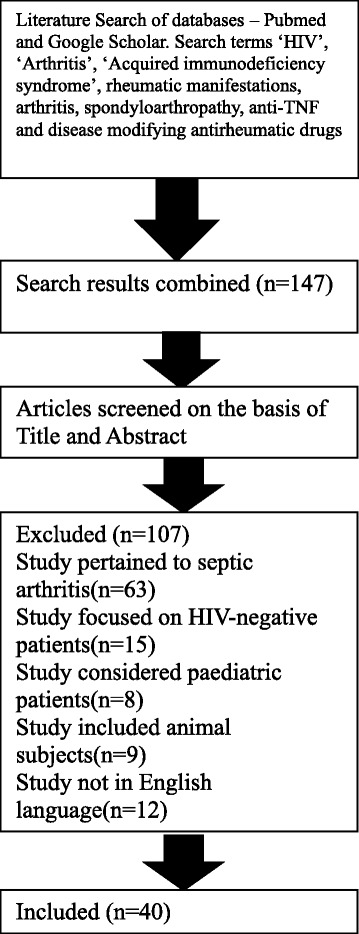
Flowchart of study identification and selection

We carried out a computer-assisted search of PubMed and google scholar for the medical literature from January 1981 to January 2015 using the keywords HIV, acquired immune-deficiency syndrome, rheumatic manifestations, arthritis, spondyloarthropathy, anti-TNF and disease modifying antirheumatic drugs. Only English language literature was included and only studies involving adult human subjects were assessed (Fig. 1).

## Results

### HIV-associated Arthropathy

HIV-associated arthritis can occur at any stage of HIV illness. It presents as an asymmetric oligo arthritis, symmetrical polyarthritis or as a monoarthritis. The asymmetric, oligo arthritis variant is the most common form, has a male preponderance, and predominately affects the knees and ankles [5]. The symmetrical polyarthritis variant closely mimics RA, with patients exhibiting similar deformities to rheumatoid patients, including ulnar deviation. It is characterised however by greater acuity at onset and is usually nonerosive. The presence of Jaccoud arthropathy as part of an HIV-associated arthritis has also been described occasionally [6]. HIV-associated arthritis tends to be short lived with its peak intensity occurring in 1 to 6 weeks [7]. However, some patients develop a chronic destructive arthropathy, associated with marked functional disability [8]. Features of mucocutaneous involvement or enthesopathy are rare. Radiological changes can occasionally mimic RA, with joint space narrowing, erosions and periarticular osteopenia [5]. However, some patients demonstrate new bone formation - a radiological finding unusual in RA [9]. There is an inflammatory, but sterile pattern on synovial fluid analysis with white cell count in the region of 50–2600 cells/μL, and normal glucose [5]. ANA, Rheumatoid Factor and HLA B27 are negative [10]. There remains considerable debate as to whether HIV infection is the cause of, or a coincidental finding in patients who are HIV positive and seronegative with an inflammatory arthritis. The evidence supporting the hypothesis that HIV has a direct inflammatory effect on synovial tissue is twofold. Firstly, p24 antigen, HIV DNA and tuboreticular inclusions have been detected in the synovial fluid of affected joints [11]. These all point to a viral aetiology. P24 antigen in particular has been found in joints at levels ten times higher than the serum levels [11]. Secondly, there is the epidemiological observation of increased prevalence of rheumatic conditions in HIV positive patients [5]. Some authors have speculated that the true prevalence of HIV-associated arthritis is higher than is generally recorded [11].

### Painful articular syndrome

The painful articular syndrome is a self-limited disorder of unknown aetiology, lasting for less than 24 h in patients with HIV infections. It is reported in up to 10 % of African HIV-seropositive patients [12] and is noted to be more common in those with advanced infection [13]. Bone and joint pain is noted, especially in the lower extremities in an asymmetric pattern, which is out of proportion to clinical findings. Though synovitis is absent, pain is excruciating and debilitating leading to hospital treatment in more than half of patients. The joints most commonly involved are the knees, shoulders and elbows [11]. Its prevalence appears to have reduced since the advent of HAART [14].

### Spondyloarthropathies

The emergence of HIV has changed the epidemiological profile of rheumatic diseases particularly in sub-Saharan Africa. The SPA were previously described as uncommon in sub-Saharan African due to the rarity of the HLA-B27 allele, but there is an increased prevalence of PsA and reactive arthritis in Africa since the outset of the HIV epidemic [15]. Severe forms of psoriasis and PsA have been reported and are almost universally associated with HIV [16]. This is in contrast to Caucasian HIV positive patients where an increased severity but not prevalence of psoriasis and arthritis is seen [14]. There has been a change in the presentation and severity of these conditions since the widespread use of highly active anti-retroviral therapy [8], with the prevalence of PsA and reactive arthritis declining [14].

### Reactive arthritis

A link between HIV and reactive arthritis has been reported since the early stages of the HIV epidemic. The most typical presentation is that of a seronegative peripheral oligo arthritis predominantly involving the lower extremities, usually accompanied by enthesitis [11]. Mucocutaneous features are common - classically keratoderma blenorrhagicum and circinate balanitis. Skin involvement can be so extensive as to cause diagnostic confusion with PsA [11]. Urethritis occurs in similar frequency to HIV-negative reactive arthritis. Axial involvement and uveitis are uncommon, but do occur. HLA-B27 is found in 80–90 % of Caucasians with HIV-associated reactive arthritis, while studies of Africans with HIV-associated reactive arthritis have found nearly all to be HLA-B27-negative [7]. As in HIV uninfected patients, antecedent history of genitourinary and gastrointestinal infection is common. This point could hold the key to explaining the geographical differences observed in the prevalence of reactive arthritis – countries with higher rates of HIV contracted through intravenous drug use for example, rather than sexual contact, have observed lower rates of reactive arthritis [7].

### Psoriatic arthritis

Along with an increased occurrence, studies suggest that HIV-infected patients with psoriasis have more severe and persistent skin lesions with guttate, inverse and erythrodermic subtypes most common [17]. Similarly, those with joint disease generally suffer a more severe, deforming, erosive arthropathy refractory to conventional treatment [15], with worse clinical features in advanced HIV infection [13]. The onset of psoriatic arthritis in the setting of HIV frequently heralds the development of opportunistic infections [18]. The typical clinical presentation is an asymmetrical oligo- or polyarthritis, with a predilection for the lower limbs [16]. A symmetrical polyarthritis is also described with arthritis mutilans, but distal interphalangeal involvement and axial SPA patterns appear less freqeuntly [16]. Onset may be abrupt, with the development of erosions and disability within weeks. In addition, the number of joints affected tends to increase with time [19].

### Undifferentiated spondyloarthropathy

Some HIV-infected patients fail to develop the entire spectrum of clinical manifestations for disease to be classified as ankylosing spondylitis, reactive arthritis, or PsA, and are labelled as undifferentiated SPA. The predominant rheumatic manifestations exhibited in those with undifferentiated SPA include achilles tendinitis, dactylitis, low-back pain, plantar fasciitis, ankle pain and shoulder pain [11]. Manifestations of keratoderma blenorrhagicum and circinate balanitis are common however a lower frequency of uveitis and axial skeleton involvement is observed [20]. Psoriasiform skin rashes are also common and can be extensive. On magnetic resonance imaging and sonographic imaging, synovitis of the knees, extensive polyenthesitis, and adjacent osteitis are the frequent findings [21].

### Rheumatoid arthritis

The immune dysregulation inherent to HIV infection may interfere with the diagnosis of RA or mimic its clinical presentation. There have been reports of RA arising de novo in HIV positive patients as part of immune reconstitution syndrome following the initiation of anti retroviral therapy [8]. As already stated, HIV-associated arthritis can present as a polyarthritis of the small joints that mimics RA clinically and radiographically. Serological markers of RA can be found in HIV infected patients, and visa versa. Du Toit et al. found positive IgG RF and anti-CCP antibodies in 47 and 15 % of patients with HIV/AIDS respectively, although most patients had low titers [22]. In this study, the antibody titers reduced after 6 months of antiretroviral therapy and no patients developed RA at 1 year follow-up. Conversely, Li et al. found false-positive HIV serology in 16 % of their cohort of Chinese RA patients [23].

Several reports have suggested that patients with established rheumatoid arthritis experience clinical improvement after the development of immunodeficiency secondary to HIV [15] Recently, Tarr et al. observed that most HIV positive RA patients in their cohort had lower joint counts and composite disease activity scores despite stopping methotrexate therapy compared to HIV negative controls, supporting the suggestion that HIV infection improves RA disease activity [24]. Their study further highlighted difficulties in monitoring patients with inflammatory arthritis, who happen to be HIV positive. A persistently elevated ESR is a feature of HIV infection [25], and as a result, they demonstrated that the 28 joint count disease activity score (DAS-28) ESR overestimates disease activity by as much as 30 % when compared to DAS28 CRP (Table 1).

**Table 1:** Summary of clinical characteristics of Inflammatory Articular Syndromes in HIV positive patients

	Clinical characteristics		
Syndrome	HIV negative	HIV Positive	References
RA	Symmetrical small joint polyarthritis, hands and feet.	RA activity can improve with HIV and flare or arise de novo following HAART	-Reveille JD, Williams M. Rheumatologic complications of HIV infection. Best Practice & Research Clinical RheumatologyVol. 20, No. 6 -du Toit et alLack of specificity of anticyclic citrullinated peptide antibodies in advanced human immunodeficiency virus infection. J Rheumatol 2011;38:1055–60
	Positive Rheumatoid Factor and/or Anti-CCP	HIV infection itself can be associated with false positive Rheumatoid Factor and CCP	
	Extra articular manifestations such as interstitial lung disease and rheumatoid nodules	HIV Arthropathy can mimic rheumatoid clinically	
		ESR may remain persistently raised despite good disease control	
Reactive Arthritis	Seronegative peripheral oligo arthritis predominantly involving the lower extremities, usually accompanied by enthesitis. Keratoderma blenorrhagicum and circinate balanitis.	Skin involvement can be more florid than HIV –ve.	-Lawson E, Walker-Bone K. The changing spectrum of rheumatic disease in HIV infection Br Med Bull. 2012 Sep;103(1):203-21
		Psoriaform rashes can be so extensive as to cause diagnostic confusion with PsA.	
		Axial involvement and uveitis are less common than HIV –ve	
		HLA B27 commoner in Caucasians than black Africans	
Psoriatic Arthritis	Varied presentation:	Typical clinical phenotype is an asymmetrical oligo- or polyarthritis, with a predilection for the lower limbs	Njobvu P, McGill P. Psoriatic arthritis and human immunodeficiency virus infection in Zambia. J Rheumatol 2000;27:1699–702
	Inflammatory joint pain/spinal pain		
	Distal interphalangeal joint swelling, dactilytis, symmetrical polyarthritis, spondylitis, enthesitis and arthritis mutilans	Can present with an abrupt-onset florid polyarthritis, particularly in advanced HIV	
	History of Psoriasis or family history	More severe and persistent skin lesions with guttate, inverse and erythrodermic subtypes compared to HIV -ve	
		Distal interphalangeal involvement and axial SPA patterns appear less frequently compared to HIV -ve	
Undifferentiated Spondyloarthropathy	Clinical manifestations of ankylosing spondylitis, reactive arthritis, or PsA without full spectrum to be classified as any syndrome	Achilles tendinitis, dactylitis, low-back pain, plantar fasciitis, ankle pain and shoulder pain most commonly.	Mody G, Parke F. Articular manifestations of human immunodeficiency virus infection. Best Practice & Research Clinical RheumatologyVol. 17, No. 2, pp. 265–287, 2003
Painful articular syndrome	N/A	Severe bone and joint pain in the lower extremities in an asymmetric pattern.	Reveille JD. The changing spectrum of rheumatic disease in human immunodeficiency virus infection. Semin Arthritis Rheum. 2000;30(3):147
		No objective synovitis.	
		Can be debilitating	
HIV Arthropathy	N/A	Presents as an asymmetric oligo arthritis, symmetrical polyarthritis or as a monoarthritis.	Plate A-M, Boyle B. Musculoskeletal Manifestations of HIV. AIDS Read. 2003;13(2)
		Patients lack features of mucocutaneous involvement or enthesopathy	
		Symmetrical polyarthritis variant closely mimics RA.	
		Occasional erosions and joint space narrowing radiographically	
		ANA, Rheumatoid Factor and HLA B27 are negative	
		Sterile, inflammatory synovial fluid	

## Discussion

### Therapy of inflammatory arthritis in HIV positive patients

There are potential issues regarding the safety of disease modifying anti rheumatic drugs (DMARD) and biologic therapy in HIV positive patients, with most available data coming from case series. Non-steroidal anti-inflammatory drugs remain the first line treatment for HIV-associated arthritis. Due to the typically self-limited nature of the condition, DMARDS are rarely required [15]. This is in contrast with the other forms of arthropathy found in association with HIV infection. The arthritis of undifferentiated SPA, and indeed all the SPA can improve significantly with highly active antiretroviral treatment alone [26]. Both the arthritis and the cutaneous lesions of HIV-associated reactive arthritis and PsA have been found to respond to etretinate (0.5–1.0 mg/kg/day) according to one report [27].

Interestingly, both Indomethacin and hydroxychloroquine have demonstrated antiretroviral activity in small case series [28–30]. The hydroxychloroquine dose used in one series was 800 mg daily however - a dose not recommended in routine rheumatological practice. Methotrexate was initially viewed as contraindicated in HIV infection due fears of an increased risk of opportunistic infections [31], but is nowadays used cautiously in HIV positive RA and PsA patients with higher CD 4 counts (greater than 100/mm3 [11]), as long as close monitoring of cell counts is performed [24]. It is still felt though that it should be avoided in those with concomitant hepatitis C infection [11]. Sulphasalazine (at a dose of 1–2 g daily) has been successfully used to treat SPA with no clinical deterioration in HIV infection [32], and in one reported case even resulted in an improvement in CD 4 count in a patient with reactive arthritis [21]. Leflunomide at a dose of 20 mg daily (used specifically to treat HIV rather than arthritis) has also been shown to reduce HIV replication [33]. Mycophenolate (1 g BD) [34], azathioprine [35] and gold [36] have also been reported to be efficacious in HIV positive patients with PsA. Short courses of prednisolone are also considered safe, even in advanced HIV infection [37]. In one series, Bromocriptine has also been associated with suppression of acute inflammatory arthritis in four out of the five patients with reactive arthritis who failed to respond to sulfasalazine alone. The exact mechanism of its action in the context is not well understood [38].

Biologic DMARDS have been used successfully in HIV positive patients with surprisingly good safety profile. A series of 8 American HIV positive patients had anti- Tumour Necrosis Factor (anti-TNF) agents (etanercept, adalimumab and infliximab) for different rheumatic conditions (two patients with RA, three with PsA, one with undifferentiated SPA, one with reactive arthritis and one with ankylosing spondylitis) with good efficacy and no adverse effects on their HIV disease [39]. The follow-up in this study was 60 months and the use of the anti-TNF agents was restricted to those with CD4 cell count more than 200 cells/ml and viral loads of less than 60 000 copies/ml at the initiation of the therapy [39]. Clearly, these patients need screening for latent tuberculosis and vigilant monitoring for the development of tuberculosis. Most recently, ustekinamab has been used in a patient with psoriasis and PsA previously refractory to methotrexate, adalimumab, etanercept and golimumab. Significant improvement was observed in his skin and joint disease and, after 2 years of therapy, he has maintained a stable CD 4 count (above 800 mm3) and an undetectable viral load while being free of opportunistic infections [40]. There has been one report however of an HIV patient with psoriatic arthritis in whom Etanercept had to be stopped due to recurrent infections [19].

There are several important drug interactions of relevance for HIV positive patients with inflammatory arthritis. For example, ritonavir is a potent inhibitor of liver enzymes CYP3A4 (which metabolises many commonly used glucocorticoids) and CYP2D6. Ritonavir can potently increase the action and duration of action of corticosteroids and severe Cushing’s syndrome has been reported on several occasions following single intra-articular injections of triamcinolone (usually as Kenalog) for musculoskeletal disease [13]. Therefore triamcinolone preparations should not be administered to patients on ritonavir.

## Conclusions

There are challenges in the management of inflammatory arthritis in patients who are HIV-positive, including difficulties in the assessment of disease activity and limited information on the safety of immunosuppressive drugs in these individuals. Registries for prospective follow up of HIV positive patients with arthropathies are urgently needed to shed light on clinical features and natural history of these conditions, and to develop treatment guidelines.

## Abbreviations

AIDS: Acquired immunodeficiency syndrome
anti-TNF: Anti- Tumour Necrosis Factor
ARV: Antiretroviral
DMARD: Disease modifying anti rheumatic drugs
HIV: Human immunodeficiency virus
PsA: Psoriatic arthritis
RA: Rheumatoid arthritis
SPA: Spondyloarthropathies
